# Supplementary material for: Avoiding data loss: Synthetic MRIs generated from diffusion imaging can replace corrupted structural acquisitions for freesurfer-seeded tractography
Source: PLoS One. 2022 Feb 18;17(2):e0247343. doi: 10.1371/journal.pone.0247343 (PMC8856573; doi:10.1371/journal.pone.0247343)
Supplement: S1 File — (ZIP) [file pone.0247343.s001.zip › SupportingInformation/SupportingInformation.docx]

# Supporting Information

Table S1. Dice Coefficients for Freesurfer’s Tissue Segmentation. Values reflect the segmentations from the in-vivo structural versus the segmentations from either the synthetic structural or in-vivo-repeat structural. Values are displayed as mean ± standard deviation. Generally, synthetic and in-vivo repeat runs produced comparable dice coefficients for white matter and CSF. For the HCP-S, the grey-matter Dice score was also comparable between in-vivo and synthetic images.

|  |  | White Matter | Grey Matter | CSF |
| --- | --- | --- | --- | --- |
| HCP-M | In-vivo Repeat | 0.92 ± 0.04 | 0.87 ± 0.07 | 0.92 ± 0.03 |
|  | Synthetic | 0.88 ± 0.03 | 0.79 ± 0.08 | 0.88 ± 0.03 |
| HCP-S | In-vivo Repeat | 0.82 ± 0.17 | 0.76 ± 0.18 | 0.83 ± 0.21 |
|  | Synthetic | 0.86 ± 0.02 | 0.74 ± 0.02 | 0.84 ± 0.02 |
| Hospital | Synthetic | 0.84 ± 0.02 | 0.71 ± 0.04 | - 1. ± 0.01 |
