# Supplementary material for: Avoiding data loss: Synthetic MRIs generated from diffusion imaging can replace corrupted structural acquisitions for freesurfer-seeded tractography
Source: PLoS One. 2022 Feb 18;17(2):e0247343. doi: 10.1371/journal.pone.0247343 (PMC8856573; doi:10.1371/journal.pone.0247343)
Supplement: Attachment — Submitted filename: responseToReviewer_v06.pdf [file pone.0247343.s002.pdf]

Dear Dr. Pham,

We would like to thank you for considering publishing our paper entitled *"Avoiding Data Loss: Synthetic MRIs Generated from Diffusion Imaging Can Replace Corrupted Structural Acquisitions For Freesurfer-Seeded Tractography"* in *Plos One*. The authors would also like to thank the reviewers for their suggestions, which have improved the quality of the manuscript. Overall, we have taken into account all Reviewers' comments, performed additional analysis and have accordingly added results in the manuscript that could be of interest for the reader.

We would like to clarify that the *"Advance Queensland Research Fellowship"* is a government grant that is not linked to any commercial source. Therefore, we have not updated the Competing Interest Statement since there was no commercial source funding this work. However, if any other modification has to be applied to the Competing Interest Statement to meet the *Plos One* requirements, please let us know and we will update this statement as required.

We hope you find this revised version of the manuscript suitable for publication in the journal *Plos One.* Please note that all the differences in the manuscript to the version originally handed in are highlighted in yellow.

Comments from Dr. Pham:

Thank you for submitting your manuscript to PLOS ONE. After careful consideration, we feel that it has merit but does not fully meet PLOS ONE's publication criteria as it currently stands. Therefore, we invite you to submit a revised version of the manuscript that addresses the points raised during the review process. In particular, all reviewers raised concerns about the need for quantitative data in the evaluation of the method. In addition, it was suggested that several relevant papers should be cited and discussed.

We are happy to include additional quantitative results in the manuscript that further assess the performances of our proposed method. Therefore, we have added zero-normalized cross correlation, generalized dice similarity coefficients, and mean diffusivity measurements to respectively provide more information about the similarity between the *in-vivo* and synthetic images, their parcellation overlap and the similarity between the diffusion measurements obtained from resulting tractography. We also referred to more relevant papers as requested by the reviewers and added information in the introduction to facilitate the manuscript understanding. Please find below the detailed response to all of the reviewers comments.

Reviewer #1:

The authors have presented and demonstrated a method to synthesize structural MRI data which would allow for increased use of scanning time for diffusion MRI data particularly in populations who are prone to movement during the scan.

1. One of the potential advantages of this method is that it could be used in a scenario with participants who move during the scan. Presumably there would be motion during the diffusion scan which could be corrected during processing. In the datasets presented in the paper how many volumes had to be excluded due to motion? Does motion affect the ability to generate the synthesized structural image and if so, to what extent?

Our method does not look at the individual diffusion-encoded volumes, but instead relies upon results produced by single-shell 3-tissue (SS3T) or multishell-multitissue (MSMT) methods, produced by the MRtrix team. These methods, in turn, rely upon the constrained spherical deconvolution (CSD) model. The CSD model's ability to handle "scrubbed" data has previously been published in some detail [Tournier, Neuroimage, 2007]. In short, at a typical b-value of 3000s/mm^2, at least 45 directions are required (fewer with lower b-values). This requirement is also cited in the SS3T paper, while the MSMT paper demonstrates its performance with a variety of different direction + shell combinations.

We feel it is outside our intended scope to formally re-assess the performance of the SS3T and MSMT models, particularly given the publications already existing on this topic. We have, however, been more explicit in our introduction about the requirements of these methods and given concrete examples of how often these are not met. For example, we have added the following:

> *Although less common, it is also possible that structural data can be corrupted, collected incorrectly, or simply lost. In such situations, the loss of structural images can completely preclude the analysis of the diffusion data using standard tools. As a concrete example, the constrained spherical deconvolution model [17] is commonly used for tractography and requires 45 uncorrupted diffusion encoding directions (assuming b=3000s/mm^2) [18,19]. This means 15 motion-affected volumes (25% of the acquired volumes) can be safely deleted from a classic 60-direction single-shell scan and the data remain analysable. We have previously reported on 123 scans of children and adolescents, the majority of whom had disability [20,21]. Reviewing these data, 63% of scans demonstrated motion artefacts in the diffusion scan but only 3.3% had 15 or more motion-affected volumes out of the 60 acquired. By contrast, 25% of structural scans displayed artefacts that precluded both structural and atlas-driven diffusion analyses [16].*

We have also added the following to the Discussion:

> *Our proposed method is dependent on the 'single-shell 3-tissue' (SS3T) or 'multishell-multitissue' (MSMT) diffusion models, which in turn are reliant on an adequate number of diffusion directions being available. We have not explored the effects of progressively reducing the number of directions to simulate movement because the requirements of SS3T and MSMT have already been explored [18,19,22]. Furthermore, our method has no additional requirements over those of typical tractography that uses constrained spherical deconvolution (at least 45 directions at a b-value of 3000s/mm^2). If these requirements are not met high-quality tractography will not be possible, regardless as to whether an in-vivo or synthetic structural is used.*

2. Were there comparisons of fiber tract orientation or other tensor based metrics in addition to using FA as a quantitative metric to assess the quality of tractography using a synthetic structural image?

We have also now included MD measurements to complement the FA measurements. These showed the same broad pattern of results. Namely, differences between pipelines (*in-vivo*, *in-vivo* repeat, and synthetic) were not statistically significant and their amplitudes were below

1% of the ground truth measurement, meaning the differences have little to no practical significance.

A relevant paper to cite is by Hu Cheng et al (June 2020) titled "Segmentation of the brain using direction-averaged signal of DWI images. Adding this to the discussion would place the proposed method in context of current alternative approaches.

We apologise for missing this reference – it was not publicly available until after our manuscript submission. We have now added this reference to our introduction, along with another which has come to light. This now reads:

> *We are aware of three previous reports in this field. Roy et al. [24] proposed a means of generating T2w images, through multi-atlas registration and patch-matching, though this method relied on the collection of adequate-quality T1w images. More simply, Dhollander and Connelly [25] normalized a single diffusion-MR derived segmentation voxel-wise and multiplied it by experimentally derived values in order to generate an image qualitatively similar to a T1w image. Similarly, Cheng et al. [26] thresholded the b0 and a mean-diffusivity-like images to identify grey-matter- and white-matter-like tissues, then applied numerical constants to combine into a T1-like image. These latter approaches both generated T1-like images, but their utility is potentially limited by relying qualitative thresholding approaches to tissue segmentations, and a lack of a physical basis for conversion to the structural image. The present work extends beyond these works by demonstrating how one can utilise established segmentation approaches that require only diffusion data and perform genuine sequence simulation that allows arbitrary T1 or T2 contrasts to be generated. We demonstrate these techniques in both healthy individuals and those with sizeable pathology.*

We would like to extend thanks to Reviewer 1 for their suggestions and taking time to review our manuscript.

Reviewer #2: This paper presents a physics based method for synthesizing T1w and T2w MR images from diffusion MR images, which first estimates tissue volume from diffusion data using multi-tissue spherical deconvolution, and then uses the estimated tissue volume for bloch simulation of different sequences such as MPRAGE. The method uses the synthesized T1w data for FreeSurfer reconstruction for brain segmentation, which is then used for delineating brain ROIs for diffusion tractography. This method is novel and interesting, and might be a potential useful tool for the neuroimaging community. The paper is clearly written, except for the intro which needs better motivation and literature review. The experiments are well designed but the evaluation of the proposed method should be more comprehensive and thorough. My detail comments are as below.

(1) The introduction for diffusion MRI and structural MRI in the first paragraph is not very friendly to non-MRI readers. Maybe provide more background. Also maybe provide more explanations on why "such analyses almost always require identification of cortical or subcortical structures using an aligned structural MR image, such as a T1- or T2-weighted scan".

We have expanded our introduction to be slightly more digestible for those who are new to the field of diffusion MRI. The opening paragraph now reads:

> *Diffusion magnetic resonance imaging (MRI) is a form of medical imaging that can indirectly quantify certain aspects of tissue microstructure related to myelination [e.g. 1], axon density [2], or cellular death. It also can, via tractography, delineate axonal pathways from which such microstructural measurements can be taken. These abilities make diffusion MRI a popular means of identifying and quantifying brain reorganisation and injury [3–5]. Studies utilising diffusion MRI commonly attempt to report measurements in specific white-matter tracts, or in specific regions of interest, which almost invariably requires identification of cortical or subcortical structures. Such parcellation typically relies upon an aligned structural MR image, such as a T1- or T2-weighted scan (referred to herein as 'structural images' for simplicity). This reliance is because most standard neuroimaging packages that can provide parcellations, such as Freesurfer [6], have been optimised for high-resolution images displaying particular tissue contrasts that are not apparent in diffusion images. The reliance on structural images to complete diffusion analyses can introduce four difficulties into analysis pipelines.*

(2) The first paragraph to some extent provides some motivation on why "Such reliance on structural images can introduce four 4 difficulties into analysis pipelines", which need some refinement and detailed explanation. The main issue between the co-reg between the T1w and the diffusion data is the susceptibility induced non-linear distortion present in the diffusion data. This non-linear distortion can be corrected e.g. using blip-up/blip-down b=0, but this data might be not acquired in some cases. The other main reason is that sometimes the T1w is also missing. Can refer to these papers: Little and Beaulieu. NeuroImage 2021, 237, 118105. Bhushan, Haldar, et al. NeuroImage 2015, 115, 269-280.

(3) In general, the intro is too brief which requires more details. Also the intro needs to better discuss previous similar works and do a better literature review.

We have opted to answer Questions 2 and 3 together, given their similarity. As suggested, we have now considerably expanded the Introduction to give more detailed explanations for these four difficulties, including relevant citations to Bhushan et al., as helpfully suggested by the reviewer. The introduction now not only includes substantially more detail, but is much more densely cited with previous works, including attempts to tackle the major problems stated and two previous attempts to synthesise structural images. We include a discussion as to why our method goes beyond these previous attempts. Our edits are too numerous to list here, but we encourage the reviewer to see them in the submitted updated manuscript.

(4) The paper only qualitatively compares the synthesized images and the actually acquired T1w or T1w images. Maybe consider comparing them quantitatively using metrics such as SSIM using up-sampled diffusion synthesized images.

The contrast between two given pure tissues, as measured in the current study, is a quantitative measure of the MR pulse sequence signal response according to the magnetic properties of the two given tissues. Therefore, if brain tissue contrasts are similar between synthetic and *in-vivo* images, this quantitatively shows that the model employed to generate the synthetic image provides a similar signal response to the MR pulse sequence used for the *in-vivo* image acquisition. However, we agree that the contrast provides no quantitative information about the spatial similarity between *in-vivo* and synthetic images.

While the tissue contrasts are quantitative, the absolute intensities of *in-vivo* T1w and T2w images are qualitative by nature. In other words, the same scan performed twice on the same subject can produce images with different intensity ranges (though similar tissue contrasts). The SSIM is inappropriate for comparing non-quantitative scans as it punishes images acquired with different intensity ranges. Non-destructive intensity normalization (such as subtracting the image mean and dividing by the variance) can be performed to overcome this, but to some degree this defeats what the SSIM was designed to measure. Instead, we computed the zero-normalized cross-correlation as it provides quantitative information about image similarities without requiring the images to lie in the same intensity range. The details about the zero-normalized cross-correlation computations and the results obtained were added to the manuscript. We also included a brief comment on why standard similarity metrics such as the SSIM could not be used in the current study. The update in the materials and methods section is as follows:

> *The zero-normalized cross correlation was computed to further assess the similarity between the in-vivo and synthetic structural scans. This metric was preferred over standard similarity metrics, such as the structural similarity index, as it does not require that the images being compared have the same intensity range (T1w MPRAGE and T2w spin-echo contrasts are qualitative, and so repeat scans and synthetic images do not necessarily lie in the same intensity range as the first in-vivo image). To allow for the computation of the zero-normalized cross correlation, the synthetic images were spatially normalized to the in-vivo images using the affine registration tool provided by ANTS 2.1 [42]. The brain masks computed in the diffusion image space (see section 3.2.2) were then propagated in the structural image space to skull-strip the in-vivo images. The zero-normalized cross-correlation was computed as follows:*

The results section of the manuscript was also updated as follows to include the zero-normalized cross-correlation measurements:

> *A very strong zero-normalized cross-correlation was measured between the co-registered T1w in-vivo and synthetic images for the Hospital (mean: 0.87; range: 0.82-0.90), HCP-M (mean: 0.86; range: 0.84-0.87) and HCP-S (mean: 0.86; range: 0.83-0.88) datasets. The zero-normalized cross-correlation measured between the co-registered T2w in-vivo and synthetic images was strong for both HCP-M (mean: 0.75; range: 0.67-0.80) and HCP-S (mean: 0.68; range: 0.64-0.73) datasets.*

(5) Even though the brain segmentation is the main focus of the method, there is no evaluation or comparison of the FreeSurfer results from synthesized images and actual images, which is much easier to perform than for images. Figure 3 only shows tissue and cortical segmentation visually. The Dice coefficients or some overlapping metrics for the segmentation of each

cortical area and each brain region from synthesize and actual images should be reported to quantify the performance of the proposed method. The segmentation from the diffusion synthesized images can be upsampled using nearest neighbour resampling.

We are unclear as to whether the reviewer is implying the focus of the manuscript is tissue segmentation or regional parcellation (identification of brain structures). To clarify, our focus is to enable the use of tools such as Freesurfer for *parcellation* – tissue segmentation is already relatively easy to perform with diffusion images without needing structural images at all (e.g. Mrtrix' SS3T and MSMT methods that we utilised here). The goal of such parcellation is only to support fairly classical diffusion analyses, such as region-to-region tractography. With this in mind, the performance of parcellation is only relevant with regards to how it affects the final diffusion metrics. For example, let us suppose the Dice metrics for Freesurfer are slightly degraded in the synthetic scan but near the pial surface. If this is the case, the Dice metric will be lower but the resulting tractography will be unaffected. This is why we originally focussed on the final tractography metrics, as well as Dice metrics of the tractography itself.

Nevertheless, recognising that we cannot possibly cover all possible white-matter anatomies, tracking methods, tractography settings, and so on, we have expanded our results to include Dice coefficients for regional parcellations. The tissue segmentation Dice coefficients remain in the Supplementary Materials, as per the original manuscript, with a much clearer reference within the main manuscript highlighting their inclusion.

As a summary, Dice scores were similar between *in-vivo* and synthetic pipelines for the 2mm isotropic images. At 1.25mm, the *in-vivo* repeat scans had higher dice scores than the synthetic pipeline, but the worst-case performance of the two methods was equivalent. We have framed this in the discussion as an indication that with high-resolution (1.25mm) diffusion data an *in-vivo* scan is preferable where parcellation accuracy is important, but in the case of data loss (the issue we are attempting to solve), the synthetic image can still prove a worthwhile backup. An extract of this discussion reads as follows:

> *We also calculated generalised Dice coefficients (gDSC) for 70 cortical GM labels and 16 deep GM labels, at the GM/WM interface (**Error! Reference source not found.**). For the HCP-S dataset, gDSC scores for the synthetic and in-vivo repeat pipelines were comparable. Results for the HCP-M dataset, however, implied that groups who work with high resolution (1.25mm) diffusion data whose pipelines rely on a particularly accurate cortical parcellation would do best to rely on an in-vivo structural image (if available), rather than a synthetic structural image.*
>
> …
>
> *Notably, the worst-case performance was equal between the synthetic and in-vivo pipelines for high-resolution (HCP-M) diffusion data, while at the more typical resolution of 2mm (HCP-S), the synthetic pipeline had better worst-case performance than the in-vivo pipeline. Such results highlight that while a genuine structural image is usually preferable, when this is lost or corrupted a synthetic image can act as a worthwhile substitute.*

(6) Showing and comparing tractography results on only one tract, i.e., superior thalamocortical tract, is not sufficient. Results from more tracts need to be reported and ideally the whole-brain structural connectivity should be reconstructed and compared. The reconstruction of the structural connectivity requires the use of each segmented brain region from the synthesized images, and therefore could more accurately and comprehensively reflect the quality of the synthesized images and their FreeSurfer reconstruction results.

We understand the suggestion to perform even more tractography, though we feel that this is a goal that can snowball very quickly. Connectomes can be seeded, tracked, filtered, and clustered in dozens of ways, and with several different libraries and atlases, all of which can have substantial effects on the final result (for example, see Messe, HBM, 2019). The reproducibility of connectomes also requires numerous metrics to make a fair comparison (for example, see Bonilha, Plos One, 2015), all of which are affected by factors such as resolution (e.g. see Reus, NeuroImage, 2013).

Given the variability in how connectomes are performed, we feel that to attempt a reliability measurement here would not be helpful to many individual readers due to methodological mismatches, while simultaneously increasing the size of the paper greatly. We note that the results section of latest revision now includes three sections and around seven pages of results, including: qualitative and quantitative image similarity measures; Dice of two superior thalamocortical tracts; FA and MD measurements of these tracts; Dice coefficients of cortical labels, and sub-cortical labels; and Dice-coefficients for GM, WM, and CSF (in supplementary materials).

Importantly, this latest revision now provides generalised Dice coefficients for cortical labels at the grey-matter/white-matter interface – the voxels that ultimately have influence on all connectomes – and note that at the more-common resolution of 2mm there were not statistically significant differences between *in-vivo* and synthetic pipelines. We hope that such results are considered sufficient to guide most readers who work with connectomes as to whether the proposed method is likely to be a worthwhile alternative to simply excluding participants when structural images are lost.

We would like to extend our thanks to Reviewer 2 for taking time to provide us with honest and robust feedback that has helped us to improve our paper.

Reviewer #3: Authors present an approach for generating synthetic T1w and T2w image from diffusion MRI images by using Bloch equations along with tissue partial volume estimates from diffusion MRI. The synthetic images are used in a diffusion tractography pipeline. The method is clearly described and results are presented on HCP and a private hospital data. In my opinion, the results do not provide evidence of robustness to nuisances and repetition of diffusion MRI acquisition. Also, the method assumes very simple tissue model parameter (table 1) that is assumed to be constant across population and throughout the brain, which will most likely be not true in scenarios "where structural image corruption is common, such as studies of children or cognitively impaired persons". My specific comments are below:

- Same relaxometry value (table 1) is very simple. These values will most likely not match in children and subjects with cognitive impairment. Please comment and, if possible, include some results in children.

Since the synthetization of the T1-weighted and T2-weighted images is based on the solutions of the Bloch equations, the relaxometry values employed are considered as input variables to the synthetization model. Therefore, these relaxometry values can easily be adapted to the population of interest to synthesize T1- and T2-weighted images with a contrast corresponding to the contrast that is expected to be obtained *in-vivo* for the population of interest. However, our synthetization model is likely to not work on neonates as the GM/WM/CSF synthetization approach is designed to model tissues that have not yet fully developed in the neonate brain. We have added the following comment about the adaptability of the method in the discussion:

> *One limitation of the current approach is the assumption that the brain tissues have the same relaxometry values for all subjects, while the subject relaxometry values may change due to the subject age or brain disease. However, the relaxometry values are used as input variables in the solutions of the Bloch equations for the synthetization of the structural images, thus allowing to easily tailor the model by using relaxometry values corresponding to the population of interest (ie. young vs aged subjects, type of pathology, ...). Nevertheless, the proposed method is likely to not work for neonates as it models tissues that have not yet fully developed in the neonate brain.*

- Because of the simple tissue model and that fact that "specific care was taken to avoid the inclusion of partial volume voxels within the ROIs" the contrast comparison (eq 4) does not sufficiently identify quality of synthetic images. Probably a difference map between in-vivo and synthetic image would be more meaningful (and compare how it look wrt difference map between in-vivo repeats). Also, in table 2, In-vivo Hospital and Synthetic Hospital differ a lot for WM/GM.

There are two points made here which we would like to untangle. Firstly, the reviewer has suggested that the tissue contrasts are not sufficient to compare image similarities, with particular focus on avoidance of voxels containing multiple tissues. Secondly, the reviewer has suggested that contrasts for the Hospital dataset differ greatly.

With regards to the tissue contrasts, these measurements were never intended to compare the structural similarity of the two images, but only to assess the similarity between the MR pulse sequence signal response and the synthetic model signal response in two different pure tissues. Partial volume voxels were deliberately excluded from the contrast computation to ensure its measurement in pure tissues, i.e. to ensure that contrast values could be interpreted as fair metrics of the similarity between the synthetic and *in-vivo* tissue responses. If care is not taken to exclude partially-volumed voxels then these metrics become unfair comparisons, reflecting mismatched degrees of partial voluming rather than tissue contrasts *per se*. We also took care to ensure that the ROIs used for the contrast computations were physically close to one another to greatly reduce the effect of B1 field inhomogeneities on measurements. Therefore, assuming that the solution of the Bloch equations used for the simulations correctly model the MR pulse sequence signal responses (which we believe is undisputed in the literature), the difference in contrasts between the synthetic and *in-vivo* images should be mainly due to differences in synthetic and *in-vivo* relaxometry values. This means that large contrast differences would indicate that the relaxometry values used for image simulation do not correspond to the *in-vivo* relaxometry values of the population imaged. In other words, a large contrast difference implies that a "wrong" or "simple" relaxometry model is used for the simulation.

With respect to the contrast of the Hospital dataset, we are inclined to disagree with the reviewer that we reported substantially different contrast values for *in-vivo* and synthetic datasets. The contrast value ranges from 0 to 1. In absolute terms, the difference between the mean WM/GM *In-vivo Hospital* and *Synthetic Hospital* contrasts was 0.05 points. For comparison, the WM/GM contrast in the *in-vivo HCP* dataset had a range of 0.06 points. So, while there is a difference here, it is marginal when considering the range of the WM/GM contrasts seen in *in-vivo* datasets. This 0.05 point difference is also possibly exaggerated by the small differences between the MPRAGE parameters used for the *in-vivo* acquisition ($\alpha = 9°$; $TE = 2.9\,ms$; $TI = 900\,ms$; $TR = 1900\,ms$) and image synthetization ($\alpha = 9°$, $TE = 2.9\,ms$, $TI = 900\,ms$, $TR = 2300\,ms$) or by the presence of B1 field inhomogeneities. This is also visually marginal (Figure 2) and unlikely to affect most image processing packages. In our study, for example, Freesurfer was able to complete processing for all synthetic datasets (excepting one, where it also failed on the *in-vivo* image).

As previously discussed in the response from Comment 4 of Reviewer 2, we agree that the contrast does not sufficiently identify the quality of synthetic MR images as it does not provide any information about the spatial similarity between the *in-vivo* and synthetic structural images. Similarly to our comments on SSIM, computing the difference between these images would not provide meaningful quantitative information as the synthetic structural images are not necessarily lying in the same intensity range as the *in-vivo* structural images, since T1w and T2w images are qualitative by nature. Instead, we computed the zero-normalized cross-correlation to provide quantitative measurements about both the spatial and intensity similarity between *in-vivo* and synthetic structural images. The following results now appear in our paper:

> *A very strong zero-normalized cross-correlation was measured between the co-registered T1w in-vivo and synthetic images for the Hospital (mean: 0.87; range: 0.82-0.90), HCP-M (mean: 0.86; range: 0.84-0.87) and HCP-S (mean: 0.86; range: 0.83-0.88) datasets. The zero-normalized cross-correlation measured between the co-registered T2w in-vivo and synthetic images was strong for both HCP-M (mean: 0.75; range: 0.67-0.80) and HCP-S (mean: 0.68; range: 0.64-0.73) datasets.*

- In all results, synthetic images are generated from same diffusion data. This does not provide evidence that the proposed method is robust to nuisances, motion and artifacts in diffusion data. It would have been specially interesting to see how it works when diffusion data is also corrupted by motion because subjects with motion in T1w/T2w images generally also have motion in diffusion. Also, its important to show repeatability of the method on diffusion data repeats.

We are a little confused with respect to the comment that our images were generated from the “same diffusion data”. We applied this technique to 32 scans, including those acquired on different scanners, with different protocols and with substantial pathology present. Presumably, this is sufficient to capture at least some spread of ‘nuisances’, differences between scanners, and differences between protocols.

The reviewer’s comment on motion artefacts is similar to that of Reviewer 1s, and our response is much the same. The large difference between structural and diffusion images is that “scrubbing” of motion affected volumes is simple in diffusion images but effectively impossible in structural images unless raw k-space is retained.

With respect to our method's robustness to motion, we would like to re-highlight that our method does not look at the individual diffusion-encoded volumes, but instead relies upon results produced by single-shell 3-tissue (SS3T) or multishell-multitissue (MSMT) methods, produced by the MRtrix team. These methods, in turn, rely upon the CSD model. Motion corrupted volumes should never be passed to CSD, but rather removed during preprocessing. The CSD model's ability to handle "scrubbed" data has previously been published in some detail [Tournier, Neuroimage, 2007]. In short, at a typical b-value of 3000s/mm^2, at least 45 directions are required (fewer with lower b-values). Even in difficult-to-scan populations, this is an easy target to reach. This requirement is also cited in the SS3T paper, while the MSMT paper demonstrates its performance with a variety of different direction + shell combinations.

We feel it is outside our intended scope to formally re-assess the performance of the SS3T and MSMT models, particularly given the publications already existing on this topic. We have, however, been more explicit in our introduction about the requirements of these methods and given concrete examples of how often these are not met. For example, we have added the following:

> *Although less common, it is also possible that structural data can be corrupted, collected incorrectly, or simply lost. In such situations, the loss of structural images can completely preclude the analysis of the diffusion data using standard tools. As a concrete example, the constrained spherical deconvolution model [17] is commonly used for tractography and requires 45 uncorrupted diffusion encoding directions (assuming b=3000s/mm^2) [18,19]. This means 15 motion-affected volumes (25% of the acquired volumes) can be safely deleted from a classic 60-direction single-shell scan and the data remain analysable. We have previously reported on 123 scans of children and adolescents, the majority of whom had disability [20,21]. Reviewing these data, 63% of scans demonstrated motion artefacts in the diffusion scan but only 3.3% had 15 or more motion-affected volumes out of the 60 acquired. By contrast, 25% of structural scans displayed artefacts that precluded both structural and atlas-driven diffusion analyses [16].*

We have also added the following to the Discussion:

> *Our proposed method is dependent on the 'single-shell 3-tissue' (SS3T) or 'multishell-multitissue' (MSMT) diffusion models, which in turn are reliant on an adequate number of diffusion directions being available. We have not explored the effects of progressively reducing the number of directions to simulate movement because the requirements of SS3T and MSMT have already been explored [18,19,22]. Furthermore, our method has no additional requirements over those of typical tractography that uses constrained spherical deconvolution (at least 45 directions at a b-value of 3000s/mm^2). If these requirements are not met high-quality tractography will not be possible, regardless as to whether an in-vivo or synthetic structural is used.*

- Because same diffusion data is used for all results, it is expected that the results will have very similar tracks and FA values. The source of the difference are small changes in ROI seeding, which will have minor impact on the tracks. So, in my humble opinion, these results (fig 4-5) are expected because same diffusion data was used.

We are not certain we fully understand the comment here. If the reviewer is stating that results are robust when we have used different structural images but kept the diffusion datasets unchanged, then we agree. Our goal was to demonstrate that our synthetic images are worthwhile replacements when structural images are lost. As such, we processed data with the *in-vivo* and synthetic structural images and compared results. We were pleased to find (and agree) that tractography is generally robust to subtle changes in ROI locations, and thus subtle differences between synthetic and in-vivo images had negligible impact on final tractography results. This indicates that our synthetic images are worthwhile substitutes for *in-vivo* images when these are corrupted or lost.

- As the synthetic images are the only difference between these results (fig 4-5) - it is probably better to show dice and other metrics on freesurfer parcels (like thalamus etc.) than on binarised tractograms.

We have now included generalised Dice coefficients for cortical labels, and for deep grey-matter labels. As a summary, Dice scores were similar between *in-vivo* and synthetic pipelines for the 2mm isotropic images. At 1.25mm, the *in-vivo* repeat scans had higher dice scores than the synthetic pipeline, but the worst-case performance of the two methods was equivalent. We have framed this in the discussion as an indication that with high-resolution (1.25mm) diffusion data an *in-vivo* scan is preferable where parcellation accuracy is important, but in the case of data loss (the issue we are attempting to solve), the synthetic image can still prove a worthwhile backup. An extract of this discussion reads as follows:

> *We also calculated generalised Dice coefficients (gDSC) for 70 cortical GM labels and 16 deep GM labels, at the GM/WM interface (**Error! Reference source not found.**). For the HCP-S dataset, gDSC scores for the synthetic and in-vivo repeat pipelines were comparable. Results for the HCP-M dataset, however, implied that groups who work with high resolution (1.25mm) diffusion data whose pipelines rely on a particularly accurate cortical parcellation would do best to rely on an in-vivo structural image (if available), rather than a synthetic structural image.*
>
> …
>
> *Notably, the worst-case performance was equal between the synthetic and in-vivo pipelines for high-resolution (HCP-M) diffusion data, while at the more typical resolution of 2mm (HCP-S), the synthetic pipeline had better worst-case performance than the in-vivo pipeline. Such results highlight that while a genuine structural image is usually preferable, when this is lost or corrupted a synthetic image can act as a worthwhile substitute.*

- Line 256: if I understand correctly, the statistical testing is incorrectly used in these results. A failure to reject the null hypothesis in a significance test does not mean that the null hypothesis is true. Wilcoxon signed-rank test tests the null hypothesis that two related paired samples come from the same distribution. A large p-value implies that null hypothesis can not be rejected and so we can not conclude anything.

We have used a more careful wording when referring to these tests. These now read:

> *Numerical differences between the in-vivo-repeat and the synthetic Dice coefficients were not statistically significant for HCP-S or HCP-M datasets*
>
> ...
>
> *FA values sampled from tractography did not differ significantly between pipelines for the HCP-M or HCP-S datasets*

- Line 294: "... accurate grey-matter/white-matter interface, which was generally of high quality here (Figure 3)." However the image show several differences in GM/WM boundaries. Please show difference map between synthetic and in-vivo image to justify this statement.

We have reworded this slightly, and now include the aforementioned dice coefficients, which directly measure the accuracy of the GM/WM boundaries. This now reads:

> *However, this is unlikely to be of serious concern for tractography-based studies which are more reliant on an accurate grey-matter/white-matter interface, which was generally of reasonable quality (**Error! Reference source not found.**), particularly for the 2mm HCP-S dataset (**Error! Reference source not found.**).*

- Line 175: While processing with in-vivo structural images affine registration was used to co-register T1 and diffusion images (FA/b=0). It is well known that EPI distortion will be not resolved by affine registration. This might be the main issue in fig 5 (line 256-258).

The reviewer would normally be correct but here the affine registration was applied after images had completed preprocessing. As these are reverse-phase encoded images, preprocessing could correct distortions reasonably well without requiring a structural image (similar results are possible with a fieldmap, though extra steps are required). In our experience, the use of non-rigid registrations between images of the same subject provides virtually no improvement over a simple affine registration, so long as images were well-collected and preprocessed with care.

- HCP-S dataset was chosen to have b-value of 3000s/mm2. Any reasons? It is related to use of CSD? Can results be included with b-value of 1000s/mm2, which is far more common.

Shells of with a b-value < 3000s/mm^2 are suboptimal for tractography, which is the primary form of analysis we are looking to support. While b-values of 1000s/mm2 are common in clinical scenarios, this is because clinics normally are focussed on simple metrics like FA and MD rather than tractography. Such low b-values are suboptimal for tractography as they fail to resolve crossing fibres. Single-shelled images are also becoming less common as they cannot be used with more modern metrics such as NODDI or AFD.

- There are some similarity of the presented method to following works. Authors should consider highlighting differences:

Bhushan C et al. "Co-registration and distortion correction of diffusion and anatomical images based on inverse contrast normalization." NeuroImage. 2015;115:269–280.

Roy, Snehashis, et al. "Patch based synthesis of whole head MR images: Application to EPI distortion correction." International Workshop on Simulation and Synthesis in Medical Imaging. Springer, Cham, 2016.

We thank the reviewer for these helpful references. They have been included in the text as suggested.

Minor points:

- "... structural images have been corrupted." However approach does not fix corruputed images but just replaces them. It abstract and introduction gives an impression that corrupted images are fixed. It should be clarified.

We have reworded this slightly. We hope the sentence in full now unambiguously states that images are being generated, not fixed:

> *To enable diffusion-image processing when structural images have been corrupted, we propose a means by which synthetic structural images can be generated from diffusion MRI.*

- DWIs are also structural images. https://jnnp.bmj.com/content/75/9/1235

We agree that DWIs can be thought of as structural images. In the context of a diffusion paper, though, it is typical to refer to T1, T2, PD and FLAIR images as 'structural' to contrast them with 'diffusion' or 'microstructural' images to avoid having to write out their names in full each time they are used. We have slightly reworded the abstract to make this distinction. We have also now explicitly stated in the introduction that 'structural images' refer to T1w and T2w images:

> *Such parcellation typically relies upon an aligned structural MR image, such as a T1- or T2-weighted scan (referred to herein as 'structural images' for simplicity).*

We would like to thank Reviewer 3 for the time spent on giving us a feedback that has helped us to improve our paper.

Sincerely yours.

On behalf of all authors,

Jeremy Beaumont,
LTSI, Campus de Beaulieu,
Université de Rennes 1,
35042 Rennes, France
Email : jeremy.beaumont.2@gmail.com
Phone number : +33 633 905 869
