# Supplementary material for: Avoiding data loss: Synthetic MRIs generated from diffusion imaging can replace corrupted structural acquisitions for freesurfer-seeded tractography
Source: PLoS One. 2022 Feb 18;17(2):e0247343. doi: 10.1371/journal.pone.0247343 (PMC8856573; doi:10.1371/journal.pone.0247343)
Supplement: Attachment — Submitted filename: responseToReviewer2_v02.docx [file pone.0247343.s003.docx]

Dear Dr. Pham,

We would like to thank you for considering publishing our paper entitled *“Avoiding Data Loss: Synthetic MRIs Generated from Diffusion Imaging Can Replace Corrupted Structural Acquisitions For Freesurfer-Seeded Tractography”* in *Plos One*. The authors would also like to thank the reviewers for their new minor suggestions, which have further improved the quality of the manuscript. Overall, we have taken into account all Reviewers’ comments and have accordingly added statistical results in the manuscript that could be of interest for the reader.

We hope you find this revised version of the manuscript suitable for publication in the journal *Plos One.* Please note the newly submitted manuscript contains yellow highlighting to indicate changes from the previous manuscript version. In this new manuscript version you may notice our office location has changed: we would like to highlight this is simply a change of physical address; our affiliations remain the same.

Reviewer #3:

*I thank authors for updating the manuscript and clarifying several issues in the last version. In my humble opinion following clarifications before publication will be valuable to the readers:*

*1. Based on the authors' responses and edits, it seems the intent and the ultimate goal of the proposed method is to obtain a synthetic T1w image that allows using existing DWI processing pipelines (like freesurfer) when an in-vivo T1w is absent. If so, I would suggest to further clarify this in abstract and introduction (Edits clarify it better in newer version, but can be further clarified). The earlier version gave impression that authors are suggesting a method that can replace in-vivo T1w/T2w images for all purposes.*

Our intent is indeed to obtain a synthetic T1w image allowing for DWI processing when an *in-vivo* T1w image is absent or motion corrupted. As suggested, we have clarified this point in both the abstract:

*To enable diffusion-image processing when structural images are missing or have been corrupted, we propose a means by which synthetic structural images can be generated from diffusion MRI.*

as well as in the introduction:

*In this study we demonstrate a straightforward means of generating synthetic T1w and T2w images from a typical diffusion acquisition to allow for diffusion MRI analysis when in-vivo T1w and T2w images have been lost or motion corrupted.*

*2. Can some statistical tests be added for results shown in table 2 and 3? This will help further strengthen the proposed method.*

We have now included p-values obtained from Bonferroni-corrected Wilcoxon signed-rank test in both Tables 2 and 3. We also updated the results section of the manuscript accordingly:

*The quantitative brain tissue contrast measurements obtained for both T1w and T2w synthetic images were close to their in-vivo counterparts for the HCP-M, HCP-S and Hospital datasets (Tables 2 and 3). The brain tissue contrasts were not significantly different between in-vivo and synthetic images (Tables 2 and 3), except for the T1w WM/GM contrast in the Hospital dataset (Bonferroni-corrected Wilcoxon signed rank test, p=0.02) and the WM/CSF and GM/CSF contrasts in the HCP-M dataset (Bonferroni-corrected Wilcoxon signed rank tests, both p=0.04). The contrast difference between in-vivo and synthetic images remained very small for these cases (Tables 2 and 3).*

*3. Line 363-365: Authors mention statistical results for HCP-S dataset (in-vivo vs synthetic ). How about statistical tests of HCP-M dataset? Median of 0.59 vs 0.75 seems to imply substantial differences.*

Response to this question appears below Comment 5.

*4. Also, please report/add the statistical test results in table 4 and supplemental material as well.*

Response to this question appears below Comment 5.

*5. Please report actual p-values of each statistical tests instead of just mentioning "P>0.05".*

We believe that there may have been confusion regarding the p-values for lines 363-365 (Comment 3) as the p-values reported are the ones from the HCP-M dataset. This confusion probably arose from the fact that the HCP-S dataset p-values were not explicitly reported, with just a mention stating that they were above 0.05 (Comment 5). We are now stating explicitly these p-values in the manuscript and Table 4 and hope that it addresses Comments 3, 4, and 5. The updated manuscript now reads:

*For the HCP-M dataset, median gDSCs were significantly lower for synthetic images than repeat in-vivo data (p=0.01 for both comparisons using uncorrected Wilcoxon Signed-Rank tests). The worst-case performances of in-vivo and synthetic pipelines were similar on this dataset. For the HCP-S dataset, differences between in-vivo and synthetic pipeline gDSCs were not statistically significant (p=0.65 for cortical GM and p=0.51 for deep GM using uncorrected Wilcoxon Signed-Rank tests).*

The missing detailed results from Table 4 have now also been included in supplemental material.

We would like to extend thanks to Reviewer 3 for their new suggestions and taking time to further review our manuscript.

Yours Sincerely,

On behalf of all authors,

Jeremy Beaumont,
LTSI, Campus de Beaulieu,
Université de Rennes 1,
35042 Rennes, France
Email : [jeremy.beaumont.2@gmail.com](mailto:jeremy.beaumont.2@gmail.com)
Phone number : +33 633 905 869
